# Experimental Basis for the High Oral Toxicity of Dinophysistoxin 1: A Comparative Study of DSP

**DOI:** 10.3390/toxins6010211

**Published:** 2014-01-03

**Authors:** Diego A. Fernández, M. Carmen Louzao, María Fraga, Natalia Vilariño, Mercedes R. Vieytes, Luis M. Botana

**Affiliations:** 1 Department of Pharmacology, Faculty of Veterinary, University of Santiago de Compostela, University Campus, Lugo 27002, Spain; E-Mails: alberto.fernandez@usc.es (D.A.F.); maria.fraga@usc.es (M.F.); natalia.vilarino@usc.es (N.V.); 2 Department of Physiology, Faculty of Veterinary, University of Santiago de Compostela, University Campus, Lugo 27002, Spain; E-Mail: mmercedes.rodriguez@usc.es

**Keywords:** okadaic acid, dinophysistoxin-1, dinophysistoxin-2, Caco-2, intestinal permeability, trans-epithelial electric resistance, occludin, annexin V, Luminex, immunoassay

## Abstract

Okadaic acid (OA) and its analogues, dinophysistoxin 1 (DTX1) and dinophysistoxin 2 (DTX2), are lipophilic and heat-stable marine toxins produced by dinoflagellates, which can accumulate in filter-feeding bivalves. These toxins cause diarrheic shellfish poisoning (DSP) in humans shortly after the ingestion of contaminated seafood. Studies carried out in mice indicated that DSP poisonous are toxic towards experimental animals with a lethal oral dose 2–10 times higher than the intraperitoneal (i.p.) lethal dose. The focus of this work was to study the absorption of OA, DTX1 and DTX2 through the human gut barrier using differentiated Caco-2 cells. Furthermore, we compared cytotoxicity parameters. Our data revealed that cellular viability was not compromised by toxin concentrations up to 1 μM for 72 h. Okadaic acid and DTX2 induced no significant damage; nevertheless, DTX1 was able to disrupt the integrity of Caco-2 monolayers at concentrations above 50 nM. In addition, confocal microscopy imaging confirmed that the tight-junction protein, occludin, was affected by DTX1. Permeability assays revealed that only DTX1 was able to significantly cross the intestinal epithelium at concentrations above 100 nM. These data suggest a higher oral toxicity of DTX1 compared to OA and DTX2.

## 1. Introduction

**Figure 1:**
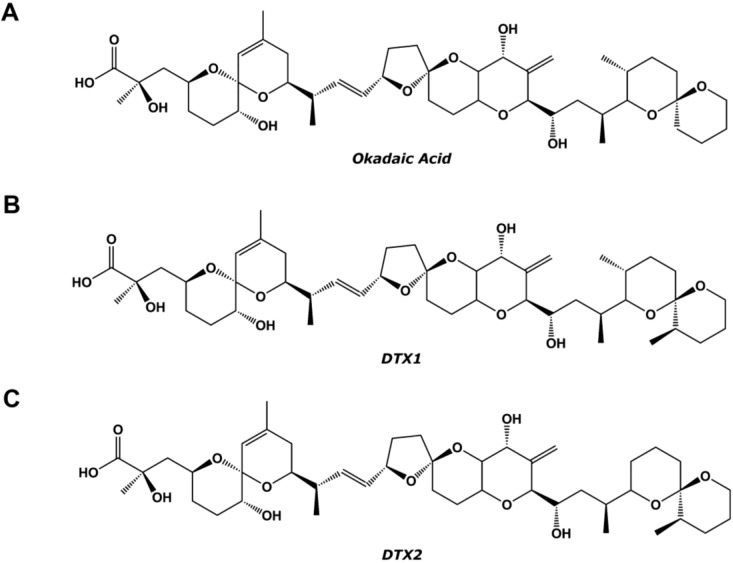
Chemical structure of (**A**) okadaic acid (OA); (**B**) dinophysistoxin-1 (DTX1); and (**C**) DTX2.

During harmful algae blooms, bivalves accumulate marine toxins, being a serious threat for human consumers of edible shellfish. Toxins causing diarrhetic shellfish poisoning (DSP) are produced by several marine dinoflagellates of the genera, *Prorocentrum* and *Dinophysis*, which are of worldwide distribution [1]. DSP is characterized by diarrhea, gastrointestinal distress, nausea, vomiting and, frequently, abdominal pain. These symptoms usually appear from 30 min to 12 h after ingestion of contaminated seafood [2]. However, as of now, there have been no records of human fatalities related to acute DSP intoxication [3]. Toxins causing DSP were first reported by Yasumoto *et al.*, in 1978 [2]. These toxins are lipophilic and heat-stable polyether compounds that can be accumulated in the hepatopancreas of filter-feeding bivalve species, such as oysters, clams, scallops or mussels [4,5]. The main compound is okadaic acid (OA); it was first isolated from the marine sponge, *Halichondria okadai*, and its structure (Figure 1) was studied in 1981 by Tachibana *et al.*, [6]. Other DSP toxins are the isomeric compound, dinophysistoxin-2 (DTX2), the methylated derivative, dinophysistoxin-1 (DTX1), and the toxins acylated at the C-7 hydroxyl group with long-chain fatty acids, collectively known as dinophysistoxin-3 (DTX3) [3,7,8,9,10]. 

The mechanism of action of this group of toxins is mainly the potent inhibition of serine/threonine protein phosphatases 1 (PP1) and 2A (PP2A) [11,12,13,14]. The different inhibitory potencies of DSP toxins on protein phosphatases have been determined allowing the establishment of inhibition equivalency factors (IEFs). The IEFs were calculated as the ratio of the IC_50_ for OA to the IC_50_ for DTX1 or DTX2. The IEF of DTX1 for PP1 is in the 0.4–0.9 range, whereas for PP2A, it is between 0.9 and 2.4 [15,16,17,18], indicating more inhibition of this phosphatase than that exerted by OA. Regarding DTX2, the IEF for PP2A is lower, with reported values ranging from 0.4 to 0.6 [18,19]. According to the current studies, the rest of the derivatives have even less inhibition effect over both phosphatases [15,20,21,22,23].

In the present work, we have studied and compared some cytotoxicity parameters of OA, DTX1 and DTX2. Our final goal was to determine the toxin absorption through the human intestine by using human colon adenocarcinoma (Caco-2) cells. 

## 2. Results

### 2.1. Cytotoxic Effect of OA, DTX1 and DTX2 in Differentiated Caco-2 Monolayers

**Figure 2:**
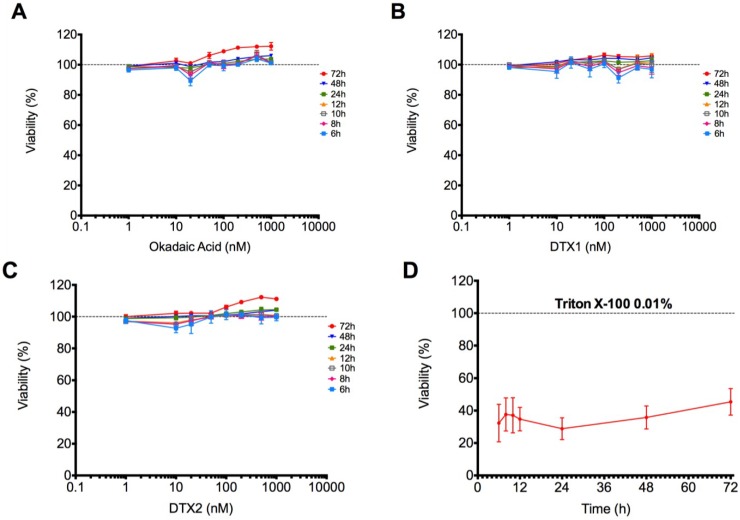
Viability of Caco-2 cells treated with OA, DTX1 and DTX2. Differentiated Caco-2 cells were incubated with increasing concentrations of (**A**) OA, (**B**) DTX1 and (**C**) DTX2 for 6, 8, 10, 12, 24, 48 and 72 h. 0.01% Triton X-100 treatment is also represented (**D**). Results are expressed as the percent of viability *vs*. control. Reported values are the mean ± SEM of three independent experiments performed in duplicate.

Metabolic activity assay: Cytotoxic effects of OA, DTX1 and DTX2 (1, 10, 20, 40, 100, 200, 500 and 1000 nM) in differentiated Caco-2 cells have been evaluated in a time-dependent manner by the AlamarBlue metabolic activity assay. Incubation with 0.01% Triton X-100 has been used as a positive control of death. Data are shown in Figure 2 as the percent of fluorescence *vs*. control. Differentiated Caco-2 cells treated with OA, DTX1 or DTX2 did not show any decrease in fluorescence intensity (Figure 2A–C). Therefore, none of the toxins affect the cell metabolism significantly, even at the highest concentration and incubation time tested (1000 nM for 72 h), indicating no change in cell viability. 

Cell membrane integrity assay: Some fluorescent methods allow a non-destructive evaluation of apoptosis by detecting changes in the structure of cell membranes. Annexin V has a high affinity for phosphatidylserine (PS), a phospholipid component located on the inner surface of the lipid bilayer of the cellular plasma membrane. During early apoptosis processes, this PS migrates to the outer portion, becomes exposed on the surface of the cell and can be detected by binding to annexin V labeled with a fluorescent dye. The annexin V assay was carried out in differentiated Caco-2 monolayers that were treated for 24 h with 100 nM of OA, DTX1, DTX2 or vehicle. Incubation with 0.005% Triton X-100 was used as a positive control of cell death and 1 μM staurosporine (STP) and 100 mM sodium butyrate (NaBT) as inductors of cell apoptosis. 

**Figure 3:**
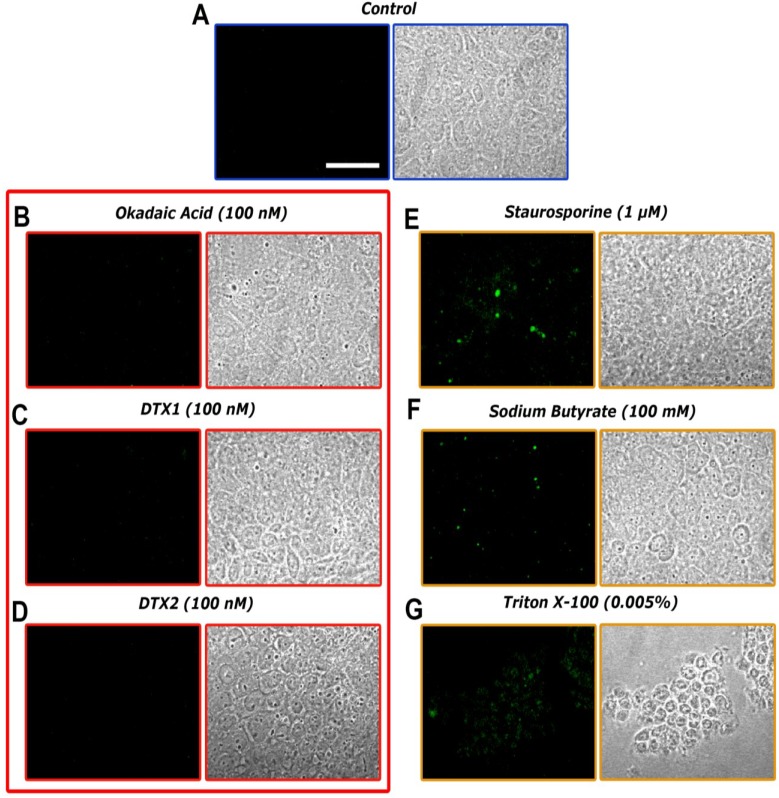
Confocal imaging of differentiated Caco-2 monolayers treated with OA, DTX1 and DTX2 and labeled with FITC-conjugated annexin V (left columns). Transmission images of the same cells are presented in the right columns. (**A**) shows control cells; while (**B***–***G**) are photographs from Caco-2 monolayers incubated for 24 h with 100 nM OA, 100 nM DTX1, 100 nM DTX2, 1 μM staurosporine, 100 mM sodium butyrate and 0.005% Triton X-1000, respectively. The images are representative of three independent experiments. Image magnification is 40×. The scale bar is 25 μm.

Control confocal microscopy images (Figure 3A) showed normal Caco-2 monolayers with no fluorescent signal, as expected. Lack of fluorescence is also found in cells incubated with OA, DTX1 and DTX2 (Figure 3B–D), revealing that no apoptotic processes were undergoing on the Caco-2 monolayers under these conditions. As for the apoptosis inductor treatments, cells incubated with 1 μM STP and 100 mM NaBT (Figure 3E,F) showed evident FITC green fluorescence zones, indicating structural changes in the plasma membrane integrity. The treatment with 0.005% Triton X-100 (Figure 3G) showed a heavily damaged cell monolayer with noticeable loss of substrate adhesion and fluorescent green dots corresponding to the FITC-marked annexin V.

### 2.2. Effect of OA, DTX1 and DTX2 on the Trans-Epithelial Electrical Resistance (TEER) of Differentiated Caco-2 Monolayers

**Figure 4:**
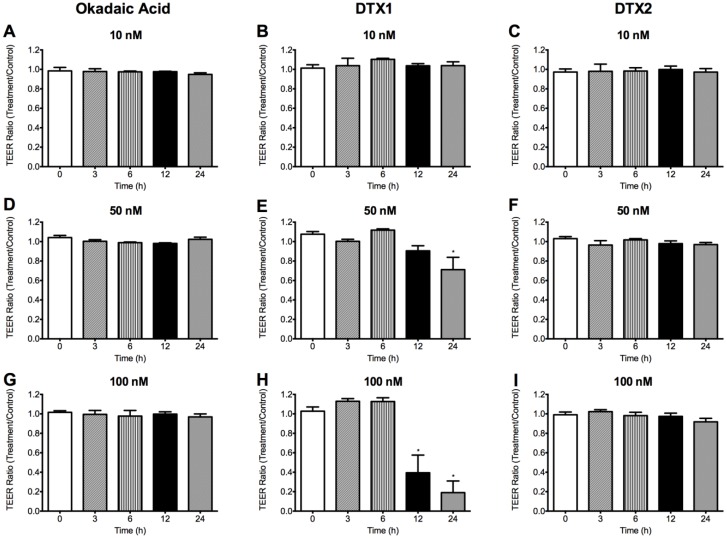
Effects of OA, DTX1 and DTX2 on the trans-epithelial electric resistance (TEER) of differentiated Caco-2 monolayers. Figure shows the TEER ratio between toxin-treated monolayers and control monolayers. (**A**,**D**,**G**) Monolayers incubated with 10, 50 and 100 nM OA; (**B**,**E**,**H**) 10, 50 and 100 nM DTX1; and (**C**,**F**,**I**) 10, 50 and 100 nM DTX2. (*) Indicates statistically different value (*p* < 0.05) *versus* time 0 h. Reported values are the mean ± SEM of three independent experiments performed in duplicate.

We assessed tight junction formation stability through measurements of trans-epithelial electrical resistance (TEER). Therefore, only inserts with TEER higher than 300 Ωcm^2^, which indicates that the Caco-2 monolayer is intact and suitable for drug permeability assays [24], were used for further testing. We studied the changes that OA, DTX1 and DTX2 induced in the TEER of the Caco-2 monolayer as a function of time. Differentiated Caco-2 monolayers were incubated with 10, 50 and 100 nM OA, DTX1 and DTX2. TEER was measured immediately before the toxins addition and after 3, 6, 12 and 24 h of incubation with them. OA and DTX2 did not induce any change in the TEER values of Caco-2 monolayers at any concentration or time tested (Figure 4). However, 50 nM DTX1 (Figure 4E) significantly decreased the TEER value when incubated for 24 h. This decrease was much more noticeable at 100 nM for 12 and 24 h (Figure 4H), indicating a clear disturbance on the Caco-2 monolayer.

### 2.3. Confocal Microscopy Imaging for Visualizing Occludin in Caco-2 Cells Treated with OA, DTX1 and DTX2

**Figure 5:**
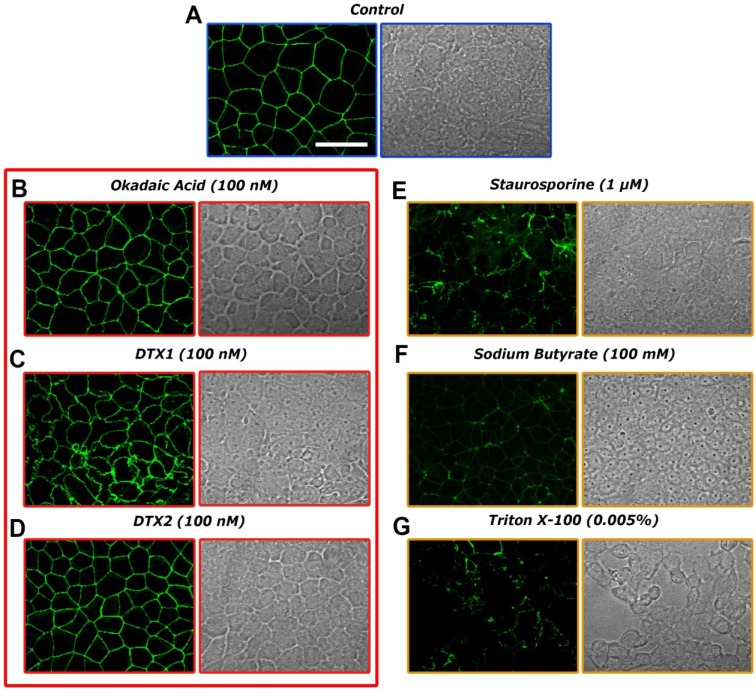
Effects of OA, DTX1 and DTX2 on occludin distribution in differentiated Caco-2 monolayers. Occludin from Caco-2 monolayers were labeled with anti-occludin, mouse monoclonal antibody (Alexa Fluor^®^ 488). (**A**) Control monolayers and monolayers incubated with (**B**) 100 nM OA; (**C**) 100 nM DTX1; (**D**) 100 nM DTX2; (**E**) 1 μM staurosporine; (**F**) 100 mM sodium butyrate; and (**G**) 0.005% Triton X-1000 for 24 h. Representative images of three independent experiments. Image magnification is 40×. The scale bar is 25 μm.

The observed decrease of TEER in the Caco-2 cells when incubated with 50 nM DTX1 for more than 12 hours suggests some damage to the monolayer that has been often linked to tight-junction disruption. Tight-junction strands are composed of membrane integral proteins, occludin being the best-studied member. We evaluate the integrity of such tight-junctions in Caco-2 cells treated with 100 nM OA, DTX1 and DTX2 for 24 h by labeling occludin. Immunofluorescent staining in control conditions (Figure 5A) showed occludin located mainly at the cell border. Neither 100 nM OA nor 100 nM DTX2 treatments (Figure 5B,D) had a significant effect on occludin location. However, Caco-2 cells incubated with 100 nM DTX1 presented fragmented and less uniform staining of occludin that even forms small vesicles at some points and showed marked indentations between adjacent cells. (Figure 5C). Treatment with 0.005% Triton X-100, 100 mM NaBT and 1 μM STP also for 24 h, which disrupted the barrier function, resulted in a redistribution of occludin with adjacent diffuse intracellular staining and a granular appearance (Figure 5E–G).

### 2.4. Permeability of Caco-2 Monolayers to OA, DTX1 and DTX2

**Figure 6:**
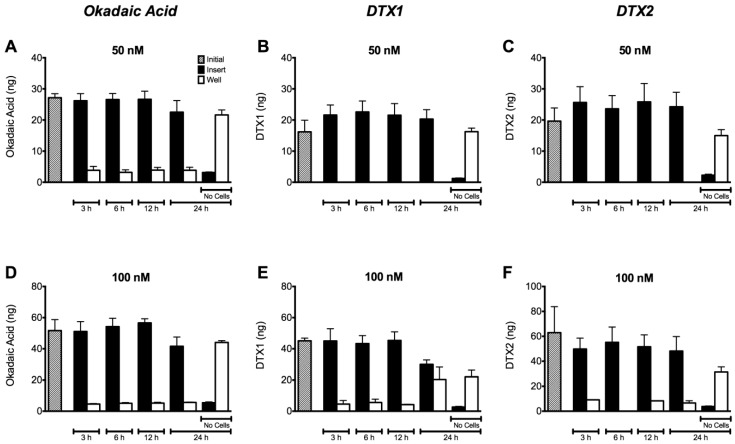
Permeability of differentiated Caco-2 monolayers to OA, DTX1 and DTX2. Toxin content in samples taken from wells and inserts with or without Caco-2 monolayers was quantified. The cells were incubated with 50 and 100 nM of (**A**,**D**) OA; (**B**,**E**) DTX1; and (**C**,**F**) DTX2. White bars show the amount of toxin in the well/receiver compartments, while black bars represent the toxin found in the inserts/donor compartments. The grey bars indicate the amount of toxin initially used in the treatments. Reported values are the mean ± SEM of three independent experiments performed in duplicate.

With the aim of evaluating the transport of OA, DTX1 and DTX2 through the cell monolayer, we performed the permeability assays as described in Section 4.7. Caco-2 cell monolayers were incubated with OA, DTX1 and DTX2 for 3, 6, 12 and 24 h. Figure 6 shows the amount of toxin quantified in samples taken from the apical (insert) and basolateral (well) side of the monolayer. According to the results, almost all the OA and DTX2 remain in the apical side (Figure 6 A,D and C,F). Thus, the toxins were unable to cross the Caco-2 monolayer under the experimental conditions used. Despite this, Caco-2 monolayers incubated with 100 nM DTX1 showed an increase in the amount of toxin in the basolateral side after 24 h of incubation (Figure 6E). DTX1 showed the highest permeability concurrent with the highest decrease in transepithelial electrical resistance (TEER) (Figure 4H). Likewise, in the absence of a cell monolayer (shown as no cells in Figure 6), all the toxins easily flow over inserts.

## 3. Discussion

At present, the Caco-2 cell line is one of the best available models of human enterocytes *in vitro*, which may represent *in vivo* intestinal epithelium well [25]. Despite their tumoral origin, these cells are able to spontaneously differentiate to small intestine enterocytes after 21 days of culture, expressing, for instance, the brush border enzyme activities [26]. In the present study, we have evaluated the cytotoxic responses of differentiated Caco-2 cells to OA and compared to the cytotoxicity of DTX1 and DTX2. OA cytotoxicity was not detected in the viability assays (Figure 2), in agreement with the data of Tripuraneni *et al*. [27] and Ehlers *et al*. [28]. In addition, our data are in accordance with Okada *et al.* [29], who detected no cell membrane damage nor LHD release with 1–2 μM OA. On the other hand, treatments with DTX1 and DTX2 up to 1 μM for 72 h did not show noticeable cytotoxic effects on differentiated Caco-2 cells. Moreover, we observed no significant apoptotic induction on cells treated with 100 nM OA, DTX1 and DTX2 for 24 h (Figure 3). 

When Caco-2 cells are cultured on inserts, they display the typical properties of an epithelial barrier, allowing for studies of permeability [30]. In these studies, TEER is a measure of the ionic movement across the paracellular pathway that can provide an indirect assessment of tight-junctions establishment and stability. Tight-junctions prevent the passage of molecules and ions through the spaces between cells. Tripuraneni *et al*. [27] found that high concentrations of OA (≥600 nM) decreased the TEER of Caco-2 monolayers, disrupting the barrier function of intestinal cells and increasing paracellular permeability in the absence of any noticeable cytotoxic response. However, Ehlers *et al.* [28] reported a lack of OA effect on the integrity of the monolayer based on the absence of TEER changes when differentiated Caco-2 monolayers were treated with up to 200 nM OA for 24h. Our results revealed that even though OA and DTX2 did not induce any change (Figure 4A,D,G and Figure 4C,F,I), 50 nM DTX1 started to decrease TEER values, lowering them to 50% after 12 h treatment with 100 nM DTX1 and to 70% after 24 h (Figure 4H). This suggests a clear effect of DTX1 on the integrity of Caco-2 monolayers when compared to the same concentrations of OA and DTX2. Nevertheless, as we could observe, a decrease in TEER does not necessarily imply cellular toxicity. Okada *et al*. in 2000 [29] also found a significant TEER reduction in Caco-2 cells in just 30 to 90 min, but with concentrations of 1 to 2 μM of OA and without cell damage or death. Wang *et al*. observed notable damage to the intestinal ultrastructure after oral administration of OA to mice, which was repaired within 24 h, which also indicate no cell death [31]. 

Thus, one of the possible reasons for the decrease of TEER in DTX1-treated Caco-2 monolayers could be the damage of tight-junctions among cells. Tight-junctions form the physical barrier to the diffusion of substances through the paracellular space [32]. Their structure consists of assemblies of trans-membrane proteins, mainly claudins and occludin [33,34], which are associated with scaffolding proteins of the zonula occludens family [35] and signaling proteins, such as protein kinases and phosphatases [36,37]. It has also been reported in the involvement of peri-junctional actin and myosin light chains in the regulation of the barrier functions of tight-junctions [33,38]. Phosphorylation and dephosphorylation processes on components of the tight-junctions, especially on occludin, have a high impact on paracellular permeability, where PP2A and PP1 seem to be key enzymes [39,40,41]. Seth *et al.* [39] reported that protein phosphatase inhibitors and reduced expression of PP2A and PP1 accelerated the calcium-induced increase in transepithelial electrical resistance and the barrier to inulin permeability and also enhanced the junctional organization of occludin and ZO-1 during tight-junction assembly in Caco-2 cells. In this context and based on our results, apart from the previously reported lack of a TEER decrease, we observed no modifications on the distribution of occludin when cells were incubated with OA and DTX2 (Figure 5B,D). Permeability assays carried out with differentiated Caco-2 cells allow us to know if the toxins are absorbed in the intestinal tract. Our results revealed no significant changes of permeability through the monolayer when the Caco-2 cells were treated with OA or DTX2 up to 100 nM for 24 h (Figure 6A,D and Figure 6C,F). Therefore OA and DTX2 are poorly transported from the luminal to the blood side when the Caco-2 monolayer is not altered. This is in line with the data generated by Ehlers *et al*. [28]; their permeability assays revealed a very limited passage of OA after 24 h with only 2% of the applied dose found in the basolateral side and about 2%–3% trapped into Caco-2 cells.

However, 100 nM DTX1-treated Caco-2 cells, whose TEER values were highly reduced, showed a clear alteration of the occludin labeling, with discontinuous distribution, indentations between adjacent cells and noticeable, numerous, rounded vesicles; opposite to the smooth and evenly distributed occludin in controls. (Figure 5C). Another possible explanation for TEER reduction could be the disruption of the actin cytoskeleton by DTX1, since the cytoskeleton is important for mechanical stability and the integrity of epithelial cells and tissues. This effect was already reported in several *in vitro* cell cultures treated with OA [29,42,43,44,45]. Some of the main domains of the plasma membrane, where actin filaments are concentrated, are substrate adhesion sites [46]. Therefore, the detachment from the substrate that DTX1 induces, on some parts of the Caco-2 monolayer (images not shown), is likely to be caused by the disruption of the actin cytoskeleton. This effect could also explain the reduction of the TEER values and the increase in permeability when the monolayer integrity has been compromised. 

The permeability of Caco-2 cells to DTX1 is significantly higher compared to the same concentration of OA and DTX2 with almost 50% of the initial amount recovered from the basolateral side at 24 h, as shown in Figure 6E. DSP toxins are potent inhibitors of Ser/Thr protein phosphatases, especially DTX1 over PP2A, but also OA over PP1 [15,16,17,18]. According to this, a 1.6-fold lower dissociation constant for DTX1 compared to OA for the catalytic subunit PP2A was reported [15]. Furthermore, a lower IC_50_ value for DTX1 with respect to OA for PP2A resulting in IEFs of 1.6–2.4 was observed [17,18]. On the contrary, different authors proposed a higher IC_50_ value for DTX1 compared to OA for the PP2A, which was translated into an IEF of 0.6–0.9 [47,48,49]. Several reasons could explain the different IEFs reported in the literature (differences in enzyme sources, enzyme concentrations, toxin standard purities, enzyme substrates and buffer compositions result in different IC_50_ values) [18]. In addition, the ability of DSP toxins to increase paracellular permeability through the inhibition of such phosphatases has been recently put into question [10]. In this study, the treatments with each toxin were performed in parallel to better establish a comparison between them. Based on the different results obtained with DTX1, OA and DTX2, we could suggest a link between the observed alteration of permeability with DTX1 and its PP2A inhibitory potency.

OA is highly toxic to mice by intraperitoneal injection, with reported LD_50_ values ranging from 192 to 255 μg/kg b.w. (body weight) for OA [6,19,50,51]. As for the acute toxicity of other DSP toxins, a minimum lethal dose of 160 μg/kg for DTX1 [5,52] and 350 μg/kg b.w. for DTX2 [19] was established. The oral LD_50_ may be 2 to 10 fold higher than the i.p. [53]. However, the data of the existing studies vary considerably. For example, Ito *et al*. [54] reported a lethal oral dose of 400 μg/kg b.w. Aune *et al*. [55] reported 880 μg/kg b.w. Tubaro *et al.* [51] reported 0/5 mice dead at 1000 μg/kg b.w and 4/5 dead at 2000 μg/kg b.w., whereas Le Hégarat *et al.* [56] found inconsistency in the results from two independent experiments with the same dose (650 µg/kg b.w.). As of now, data on the oral toxicity of OA analogs is scarce, but there are reports that suggest that DTX1 is only slightly less toxic when administered orally than when given by i.p. injection [57]. Moreover, according to Ogino *et al.* [58], the lethal oral dose of DTX1 is less than that of OA, with a reported value of 300 μg/kg b.w., suggesting higher toxicity. Therefore, the toxicity of these compounds is complex and diverse, where alterations in the tight-junction structure of epithelial cells with the addition of PP inhibition are involved in the diarrheic process. In this study, we found that DTX1 was able to reduce the TEER, disrupt the Caco-2 monolayer and pass through the intestinal epithelium. These results could further support that DTX1 is more toxic when administered orally than OA and DTX2.

The toxicity equivalent factors (TEFS) of these compounds also require attention. TEFs establishment is a matter of vital importance, especially for the analytical detection of marine and freshwater toxins. The control methods require an estimation of the toxic potential of a mixture of toxins with different potency to protect consumer’s health [18,59]. To propose the TEFs for the OA-group, EFSA assumed that all the toxins share the same biochemical mechanism of action and, therefore, were established based only on mice i.p. toxicity. TEFs are defined as the ratio of the toxic potency (usually LD_50_) for each compound compared to the potency of the reference compound (in this case, OA). These values are the following: OA = 1; DTX1 = 1; and DTX2 = 0.6 [53]. The lower inhibitory potency of DTX2 with respect to OA is comparable to its reduced acute intraperitoneal toxicity observed in mice, which allowed for the establishment of a TEF of 0.6 [19]. Nevertheless, Smienk *et al*. [49] have recently reported equal toxicity for DTX2 and OA.

Human intoxication by DSP is due to seafood consumption. Therefore, the shellfish meat regulation level of DSP toxins established by the European Union should be able to protect human health regardless of the toxin present in the shellfish sample. In this sense, our study gives new evidence that DTX1 oral acute toxicity could be higher than OA or DTX2, due to the increased absorption rate through the intestinal tract. Therefore, it constitutes a strong argument for a future revision of the actual TEF values for the analogues of the OA-group.

## 4. Experimental Section

### 4.1. Chemicals and Biological Materials

Okadaic acid (purity ≥ 98.9%), dinophysistoxin-1 (purity ≥ 98%) and dinophysistoxin-2 (purity ≥ 98%) are Certified Reference Materials (CRMs) supplied by Laboratorio CIFGA S.A. (Lugo, Spain). AlamarBlue^®^ (AB) and anti-occludin mouse monoclonal antibody labeled with Alexa Fluor^®^ 488 were purchased from Invitrogen (Camarillo, CA, USA). Trypan Blue solution 0.4%, penicillin-streptomycin solution, staurosporine (STP), Triton-X 100 and bovine serum albumin (BSA) were from Sigma-Aldrich (St. Louis, MO, USA). The Annexin V-FITC Apoptosis Detection Kit was obtained from Immunostep (Salamanca, Spain). HAM’s F-12 with stable glutamine medium, Eagle’s Minimum Essential Medium (EMEM), fetal bovine serum (FBS), gold and 18-mm Aclar coverslips were purchased from PAA (Pasching, Austria). Sodium butyrate (NaBT) was purchased from Fluka (Steinheim, Germany). Formaldehyde 16% was from Electron Microscopy Sciences (Hatfield, PA, USA). Costar 96-well assay plates with a clear bottom and tissue culture-treated surface were from Corning Inc. (New York, NY, USA), Falcon Multiwell 12-well polystyrene plates were from Becton Dickinson (Le Port de Claix, France). 12-well Milicell hanging culture inserts with a 0.4 μm pore size polyethylene terephthalate (PET) membrane were from Millipore Corporation (Billerica, MA, USA). Eight-well Nunclon plates were from Nalge Nunc International (New York, NY, USA). All other chemicals were reagent grade and purchased from Sigma-Aldrich (Madrid, Spain) and Panreac (Barcelona, Spain). Phosphate buffered saline solution (PBS) was 137 mM NaCl, 8.2 mM Na_2_HPO_4_, 1.5 mM KH_2_PO_4_, and 3.2 mM KCl, pH 7.4. 

### 4.2. Cell Lines

The Caco-2 cell line, which is derived from a human colon adenocarcinoma (ATCC N°: HTB-37), was routinely cultured in Eagle’s Minimum Essential Medium with Earle’s salts (EMEM) supplemented with 2 mM L-glutamine complemented with 10% fetal bovine serum (FBS), 1% non-essential amino acids (NEA) and 0.5% penicillin/streptomycin solution. The cells were grown in 75 cm^2^ tissue flasks incubated in a humidified atmosphere containing constant 5% CO_2_ at 37 °C. The culture medium was renewed every 2–3 days, and cells were sub-cultured by detaching them with trypsin-EDTA once per week.

### 4.3. Metabolic Activity Assay

The viability of the Caco-2 cells was measured by Alamar Blue assay, as previously described [60]. Briefly, the Caco-2 cells were seeded onto 96-well plates at an initial concentration of 25,000 cells/mL and cultured until differentiation (21 days) with medium renewal every 2–3 days. Subsequently, cells were treated with increasing concentrations of OA, DTX1, DTX2 and Triton X-100 in the presence of AlamarBlue; then, fluorescence was measured at 6, 8, 10, 12, 24, 48 and 72 hours of incubation using a FL600 Microplate Fluorescence Reader from BioTek (Winooski, VT, USA) at an excitation wavelength of 530 nm and an emission wavelength of 590 nm. Control cells were considered as having 100% viability, and the results are expressed as the percentage of the fluorescence of treated cells *versus* the fluorescence of control ones.

### 4.4. Apoptosis Detection Assay with Annexin V-FITC

To visualize apoptosis by confocal microscopy in the toxin-treated Caco-2 monolayers, an apoptosis detection kit containing annexin V conjugated with FITC from Immunostep (Salamanca, Spain) was used. 

Caco-2 cells were seeded on glass coverslips at an initial concentration of 65 × 10^3^ cells/mL and cultured for 21 days until differentiation with medium renewal each 2–3 days. On the 21st day, the cell medium was removed, and the monolayers were treated for 24 h with toxins: OA, DTX1, DTX2, vehicle and positive controls of apoptosis and cellular death (staurosporine (STP), sodium butyrate (NaBT) and Triton-X 100). Immediately after the 24 h of incubation, the medium was removed, and the cells were rinsed 3 times with PBS just before the addition of annexin V-FITC in a proportion of 1:100 diluted in the provided binding buffer. After 15 min of incubation in the dark at room temperature, the cells were rinsed again 3 times with PBS and fixed using paraformaldehyde 2% for 15 min at 4 °C. Finally, another 3 washes with PBS were required prior to mounting the coverslips over microscope glass slides with 10 μL of PBS/Glycerol (1:1) and sealing the edges with nail varnish. Confocal imaging was then carried out with a 40× oil immersion objective of a Nikon Eclipse TE2000-E inverted microscope attached to the C1 laser confocal system and EZ-C1 V.2.20 software (Nikon Instruments Europe B.V., Amstelveen, Netherlands). An argon laser was used to excite the annexin V-FITC conjugated protein (λ excitation = 488 nM, λ emission = 530 nM).

### 4.5. Trans Epithelial Electric Resistance Measurement

Trans epithelial electric resistance (TEER) was measured across differentiated Caco-2 monolayers, as previously described [60]. The measurements were performed before the treatment with OA, DTX1 and DTX2 (controls), and after each of the incubation times established: 3, 6, 12 and 24 h. Only inserts with TEER higher than 300 Ωcm^2^ before the addition of the toxin were used for further testing. 

### 4.6. Confocal Microscopy Imaging for Visualizing Occludin

Alexa Fluor^®^ 488-labeled anti-occludin mouse monoclonal antibody from Invitrogen (Camarillo, CA, USA) was used for visualizing occludin, as previously described [60]. 

Briefly, differentiated Caco-2 monolayers were treated with OA, DTX1, DTX2, vehicle and positive controls of apoptosis and cellular death: staurosporine (STP), sodium butyrate (NaBT) and Triton-X 100. Immediately after treatments, the cells were fixed and incubated with the anti-occludin antibody. Each experimental condition was performed in duplicate. Confocal imaging was then carried out with a 40× oil immersion objective of a Nikon Eclipse TE2000-E inverted microscope attached to the C1 laser confocal system and EZ-C1 V.2.20 software (Nikon Instruments Europe B.V., Amstelveen, Netherlands). An Argon laser was used to excite the Alexa Fluor 488 conjugated antibody.

### 4.7. Permeability Assay

The permeability assay was carried out as previously described [60]. During the assays, cell monolayer integrity was evaluated by measuring TEER at the start and end of the experiment. Once the incubation with the toxins (OA, DTX1 and DTX2) finished (after 3, 6, 12 or 24 h), the medium was collected from both the insert (donor compartment/apical side of the monolayer) and the well (receiver compartment/basolateral side of the monolayer) for toxin quantification purposes. Previously, we confirmed that the toxins easily flow over the naked inserts.

### 4.8. Microsphere-Based Immunoassay for DSPs Detection

OA, DTX1 and DTX2 samples taken from the inserts/donor compartments and wells/receptor compartments where quantified, as previously described by Fraga *et al.* [61]. In order to consider the cross-reactivity of the analyzed derivatives of the okadaic acid, DTX-1 and DTX-2, calibration curves were prepared using calibration solutions with CRMs of DTX-1 and DTX-2. Therefore, the quantification of DTX-1 and DTX-2 samples collected from permeability assays were quantified against their respective calibration curves. 

### 4.9. Statistical Analysis

The results were analyzed using the Student’s two-tailed *t*-test for paired data or the ANOVA 1-way test for multiple values, where appropriate. A confidence interval of 95% with a probability level *p* ≤ 0.05 was considered for statistical significance. 

## 5. Conclusions

OA, DTX1 and DTX2 did not induce any noticeable cytotoxic effects on differentiated intestinal Caco-2 cells at concentrations up to 1 μM for 72 h. However, DTX1 was able to disrupt the monolayer integrity at concentrations >100 nM for 12 h and above and to modify occludin distribution between adjacent cells. Furthermore, the permeability assay performed revealed a greater intestinal absorption of DTX1 when compared to OA or DTX2. These data suggest new evidence that the oral toxicity of DTX1 is higher than OA or DTX2. 

## References

[1] Reguera B., Velo-Suárez L., Raine R., Park M.G. (2012). Harmful dinophysis species: A review. Harmful Algae.

[2] Yasumoto T., Oshima Y., Yamaguchi M. (1978). Occurrence of a new type of shellfish poisoning in the tohoku district. Nippon Suisan Gakkaishi.

[3] Dominguez H.J., Paz B., Daranas A.H., Norte M., Franco J.M., Fernandez J.J. (2010). Dinoflagellate polyether within the yessotoxin, pectenotoxin and okadaic acid toxin groups: Characterization, analysis and human health implications. Toxicon.

[4] Food and Agriculture Organization of the United Nations (2004). Marine Biotoxins: Food and Nutrition Paper 80.

[5] Yasumoto T., Murata M. (1990). Polyether Toxins Involved in Seafood Poisoning. Marine toxins.

[6] Tachibana K., Scheuer P.J., Tsukitani Y., Kikuchi H., Van Engen D., Clardy J., Gopichand Y., Schmitz F.J. (1981). Okadaic acid, a cytotoxic polyether from two marine sponges of the genus halichondria. J. Am. Chem. Soc..

[7] Yasumoto T., Murata M., Oshima Y., Sano M., Matsumoto G.K., Clardy J. (1985). Diarrhetic shellfish toxins. Tetrahedron.

[8] Gerssen A., Pol-Hofstad I.E., Poelman M., Mulder P.P., Van den Top H.J., De Boer J. (2010). Marine toxins: Chemistry, toxicity, occurrence and detection, with special reference to the dutch situation. Toxins.

[9] Larsen K., Petersen D., Wilkins A.L., Samdal I.A., Sandvik M., Rundberget T., Goldstone D., Arcus V., Hovgaard P., Rise F. (2007). Clarification of the c-35 stereochemistries of dinophysistoxin-1 and dinophysistoxin-2 and its consequences for binding to protein phosphatase. Chem. Res. Toxicol.

[10] Munday R. (2013). Is protein phosphatase inhibition responsible for the toxic effects of okadaic acid in animals?. Toxins.

[11] Takai A., Bialojan C., Troschka M., Ruegg J.C. (1987). Smooth muscle myosin phosphatase inhibition and force enhancement by black sponge toxin. FEBS Lett..

[12] Honkanen R.E., Codispoti B.A., Tse K., Boynton A.L., Honkanan R.E. (1994). Characterization of natural toxins with inhibitory activity against serine/threonine protein phosphatases. Toxicon.

[13] Dawson J.F., Holmes C.F. (1999). Molecular mechanisms underlying inhibition of protein phosphatases by marine toxins. Front. Biosci. J. Virtual Libr..

[14] Louzao M.C., Vieytes M.R., Botana L.M. (2005). Effect of okadaic acid on glucose regulation. Mini Rev. Med. Chem..

[15] Takai A., Murata M., Torigoe K., Isobe M., Mieskes G., Yasumoto T. (1992). Inhibitory effect of okadaic acid derivatives on protein phosphatases. A study on structure-affinity relationship. Biochem. J..

[16] Holmes C.F., Luu H.A., Carrier F., Schmitz F.J. (1990). Inhibition of protein phosphatases-1 and -2a with acanthifolicin. Comparison with diarrhetic shellfish toxins and identification of a region on okadaic acid important for phosphatase inhibition. FEBS Lett..

[17] Rivas M., Garcia C., Liberona J.L., Lagos N. (2000). Biochemical characterization and inhibitory effects of dinophysistoxin-1, okadaic acid and microcystine 1-r on protein phosphatase 2a purified from the mussel *Mytilus chilensis*. Biol. Res..

[18] Garibo D., De la Iglesia Gonzalez P., Diogene J., Campas M. (2013). Inhibition equivalency factors for dinophysistoxin-1 and dinophysistoxin-2 in protein phosphatase assays, applicability to the analysis of shellfish samples and comparison with LC-MS/MS. J. Agric. Food Chem..

[19] Aune T., Larsen S., Aasen J.A., Rehmann N., Satake M., Hess P. (2007). Relative toxicity of dinophysistoxin-2 (dtx-2) compared with okadaic acid, based on acute intraperitoneal toxicity in mice. Toxicon.

[20] Hu T., Curtis J.M., Walter J.A., Wright J.L.C. (1995). Identification of dtx-4, a new water-soluble phosphatase inhibitor from the toxic dinoflagellate prorocentrum lima. J. Chem. Soc. Chem. Commun..

[21] Yanagi T., Murata M., Torigoe K., Yasumoto T. (1989). Biological activities of semisynthetic analogs of dinophysistoxin-3, the major diarrhetic shellfish toxin. Agric. Biol. Chem..

[22] Nishiwaki S., Fujiki H., Suganuma M., Furuya-Suguri H., Matsushima R., Iida Y., Ojika M., Yamada K., Uemura D., Yasumoto T. (1990). Structure-activity relationship within a series of okadaic acid derivatives. Carcinogenesis.

[23] Hu T., Curtis J.M., Walter J.A., McLachlan J.L., Wright J.L.C. (1995). Two new water-soluble dsp toxin derivatives from the dinoflagellate prorocentrum maculosum: Possible storage and excretion products. Tetrahedron Letters.

[24] Artursson P. (1990). Epithelial transport of drugs in cell culture. I: A model for studying the passive diffusion of drugs over intestinal absorptive (caco-2) cells. J. Pharm. Sci..

[25] Vignoli A.L., Srivastava R.C., Stammati A., Turco L., Tanori M., Zucco F. (2001). Nitric oxide production in caco-2 cells exposed to different inducers, inhibitors and natural toxins. Toxicol. In Vitro.

[26] Pinto M., Robine-Leon S., Appay M., Kedinger M., Triadou N., Dussaulx E., Lacroix B., Simon-Assmann P., Haffen K., Fogh J. (1983). Enterocyte-like differentiation and polarization of the human colon carcinoma cell line caco-2 in culture. Biol. Cell..

[27] Tripuraneni J., Koutsouris A., Pestic L., De Lanerolle P., Hecht G. (1997). The toxin of diarrheic shellfish poisoning, okadaic acid, increases intestinal epithelial paracellular permeability. Gastroenterology.

[28] Ehlers A., Scholz J., These A., Hessel S., Preiss-Weigert A., Lampen A. (2011). Analysis of the passage of the marine biotoxin okadaic acid through an *in vitro* human gut barrier. Toxicology.

[29] Okada T., Narai A., Matsunaga S., Fusetani N., Shimizu M. (2000). Assessment of the marine toxins by monitoring the integrity of human intestinal caco-2 cell monolayers. Toxicol. In Vitro.

[30] De Angelis I., Vincentini O., Brambilla G., Stammati A., Zucco F. (1998). Characterization of furazolidone apical-related effects to human polarized intestinal cells. Toxicol. Appl. Pharmacol..

[31] Wang J., Wang Y.Y., Lin L., Gao Y., Hong H.S., Wang D.Z. (2012). Quantitative proteomic analysis of okadaic acid treated mouse small intestines reveals differentially expressed proteins involved in diarrhetic shellfish poisoning. J. Proteomics.

[32] Pinto da Silva P., Kachar B. (1982). On tight-junction structure. Cell.

[33] Anderson J.M., Van Itallie C.M. (1995). Tight junctions and the molecular basis for regulation of paracellular permeability. Am. J. Physiol..

[34] Furuse M., Fujita K., Hiiragi T., Fujimoto K., Tsukita S. (1998). Claudin-1 and -2: Novel integral membrane proteins localizing at tight junctions with no sequence similarity to occludin. J. Cell. Biol..

[35] Al-Sadi R., Khatib K., Guo S., Ye D., Youssef M., Ma T. (2011). Occludin regulates macromolecule flux across the intestinal epithelial tight junction barrier. Am. J. Physiol. Gastrointest. Liver Physiol..

[36] Andreeva A.Y., Piontek J., Blasig I.E., Utepbergenov D.I. (2006). Assembly of tight junction is regulated by the antagonism of conventional and novel protein kinase c isoforms. Int. J. Biochem. Cell Biol..

[37] Dunagan M., Chaudhry K., Samak G., Rao R.K. (2012). Acetaldehyde disrupts tight junctions in caco-2 cell monolayers by a protein phosphatase 2a-dependent mechanism. Am. J. Physiol. Gastrointest. Liver Physiol..

[38] Turner J.R. (2006). Molecular basis of epithelial barrier regulation: From basic mechanisms to clinical application. Am. J. Pathol..

[39] Seth A., Sheth P., Elias B.C., Rao R. (2007). Protein phosphatases 2a and 1 interact with occludin and negatively regulate the assembly of tight junctions in the caco-2 cell monolayer. J. Biol. Chem..

[40] Rao R. (2009). Occludin phosphorylation in regulation of epithelial tight junctions. Ann. N.Y. Acad. Sci..

[41] Sheth P., Samak G., Shull J.A., Seth A., Rao R. (2009). Protein phosphatase 2a plays a role in hydrogen peroxide-induced disruption of tight junctions in caco-2 cell monolayers. Biochem. J..

[42] Espina B., Louzao M.C., Cagide E., Alfonso A., Vieytes M.R., Yasumoto T., Botana L.M. (2010). The methyl ester of okadaic acid is more potent than okadaic acid in disrupting the actin cytoskeleton and metabolism of primary cultured hepatocytes. Br. J. Pharmacol..

[43] Vale C., Botana L.M. (2008). Marine toxins and the cytoskeleton: Okadaic acid and dinophysistoxins. FEBS J..

[44] Vilarino N., Ares I.R., Cagide E., Louzao M.C., Vieytes M.R., Yasumoto T., Botana L.M. (2008). Induction of actin cytoskeleton rearrangement by methyl okadaate—Comparison with okadaic acid. FEBS J..

[45] Martin-Lopez A., Gallardo-Rodriguez J.J., Sanchez-Miron A., Garcia-Camacho F., Molina-Grima E. (2012). Cytotoxicity of yessotoxin and okadaic acid in mouse t lymphocyte cell line el-4. Toxicon.

[46] Small J.V., Kaverina I. (2003). Microtubules meet substrate adhesions to arrange cell polarity. Curr. Opin. Cell. Biol..

[47] Mountfort D.O., Suzuki T., Truman P. (2001). Protein phosphatase inhibition assay adapted for determination of total dsp in contaminated mussels. Toxicon.

[48] Ikehara T., Imamura S., Yoshino A., Yasumoto T. (2010). PP2A inhibition assay using recombinant enzyme for rapid detection of okadaic acid and its analogs in shellfish. Toxins.

[49] Smienk H.G., Calvo D., Razquin P., Dominguez E., Mata L. (2012). Single laboratory validation of a ready-to-use phosphatase inhibition assay for detection of okadaic acid toxins. Toxins.

[50] Dickey R.W., Bobzin S.C., Faulkner D.J., Bencsath F.A., Andrzejewski D. (1990). Identification of okadaic acid from a caribbean dinoflagellate, prorocentrum concavum. Toxicon.

[51] Tubaro A., Sosa S., Carbonatto M., Altinier G., Vita F., Melato M., Satake M., Yasumoto T. (2003). Oral and intraperitoneal acute toxicity studies of yessotoxin and homoyessotoxins in mice. Toxicon.

[52] Murata M., Shimatani M., Sugitani H., Oshima Y., Yasumoto T. (1982). Isolation and structural elucidation of the causative toxin of the diarrhetic shellfish poisoning [from the mussel mytilus edulis]. Bull. Jpn. Soc. Sci. Fish..

[53] The European Food Safety Authority (2008). Opinion of the scientific panel on contaminants in the food chain on a request from the european commission on marine biotoxins in shellfish—Okadaic acid and analogues. EFSA J..

[54] Ito E., Yasumoto T., Takai A., Imanishi S., Harada K. (2002). Investigation of the distribution and excretion of okadaic acid in mice using immunostaining method. Toxicon.

[55] Aune T., Espenes A., Aasen J.A., Quilliam M.A., Hess P., Larsen S. (2012). Study of possible combined toxic effects of azaspiracid-1 and okadaic acid in mice via the oral route. Toxicon.

[56] Le Hegarat L., Jacquin A.G., Bazin E., Fessard V. (2006). Genotoxicity of the marine toxin okadaic acid, in human caco-2 cells and in mice gut cells. Environ. Toxicol..

[57] Ito E., Terao K. (1994). Injury and recovery process of intestine caused by okadaic acid and related compounds. Nat. Toxins.

[58] Ogino H., Kumagai M., Yasumoto T. (1997). Toxicologic evaluation of yessotoxin. Nat. Toxins.

[59] Botana L.M., Vilariño N., Alfonso A., Vale C., Louzao C., Elliott C.T., Campbell K., Botana A.M. (2010). The problem of toxicity equivalent factors in developing alternative methods to animal bioassays for marine-toxin detection. TrAC Trends Anal. Chem..

[60] Fernandez D.A., Louzao M.C., Vilarino N., Espina B., Fraga M., Vieytes M.R., Roman A., Poli M., Botana L.M. (2013). The kinetic, mechanistic and cytomorphological effects of palytoxin in human intestinal cells (caco-2) explain its lower-than-parenteral oral toxicity. FEBS J..

[61] Fraga M., Vilarino N., Louzao M.C., Rodriguez P., Campbell K., Elliott C.T., Botana L.M. (2013). Multidetection of paralytic, diarrheic, and amnesic shellfish toxins by an inhibition immunoassay using a microsphere-flow cytometry system. Anal. Chem..

